# Chemically defined and small molecules-based generation of sinoatrial node-like cells

**DOI:** 10.1186/s13287-022-02834-y

**Published:** 2022-04-11

**Authors:** Xiaojie Hou, Shuhong Ma, Wei Fan, Fang Li, Miaomiao Xu, Chao Yang, Feng Liu, Ying Yan, Juyi Wan, Feng Lan, Bin Liao

**Affiliations:** 1 Department of Cardiovascular Surgery, Affiliated Hospital of Southwest Medical University, Luzhou, 646000 China; 2 Shenzhen Key Laboratory of Cardiovascular Disease, Fuwai Hospital Chinese Academy of Medical Sciences, State Key Laboratory of Cardiovascular Disease, Key Laboratory of Pluripotent Stem Cells in Cardiac Repair and Regeneration, Chinese Academy of Medical Sciences and Peking Union Medical College, Shenzhen, 518057 China; 3 Metabolic Vascular Diseases Key Laboratory of Sichuan Province, Luzhou, 646000 China; 4 Key Laboratory of Medical Electrophysiology, Ministry of Education & Medical Electrophysiological Key Laboratory of Sichuan Province, (Collaborative Innovation Center for Prevention of Cardiovascular Diseases) Institute of Cardiovascular Research, Southwest Medical University, Luzhou, 646000 China; 5 Department of Cardiology, Jianyang City People’s Hospital, Jianyang, 641499 China

**Keywords:** Human pluripotent stem cells, Sinoatrial node-like cells, Differentiation, Chemically defined medium, Small molecule, Signaling pathway

## Abstract

**Background:**

Existing methods for in vitro differentiation of human pluripotent stem cells (hPSCs) into sinoatrial node-like cells (SANLCs) require complex and undefined medium constituents. This might hinder the elucidation of the molecular mechanisms involved in cardiac subtype specification and prevent translational application. In our study, we aimed to establish a chemically defined differentiation methods to generate SANLCs effectively and stably.

**Methods:**

We induced human embryonic stem cells (hESCs)/induced PSCs (hiPSCs) to pan-cardiomyocytes by temporal modulation of the WNT/β-catenin (WNT) signaling pathway with GSK3 inhibitor and WNT inhibitor. During cardiac mesoderm stage of the differentiation process, signaling of WNT, retinoid acid (RA), and fibroblast growth factor (FGF) was manipulated by three specific molecules. Moreover, metabolic selection was designed to improve the enrichment of SANLCs. Finally, RT-PCR, immunofluorescence, flow cytometry, and whole cell patch clamp were used to identify the SANLCs.

**Results:**

WNT, RA, and FGF signaling promote the differentiation of hPSCs into SANLCs in a concentration- and time window-sensitive manner, respectively. Synergetic modulation of WNT, FGF, and RA signaling pathways enhance the pacemaker phenotype and improve the differentiation efficiency of SANLCs (up to 45%). Moreover, the purification based on lactate metabolism and glucose starvation further reached approximately 50% of SANLCs. Finally, the electrophysiological data demonstrate that cells differentiated with the proposed protocol produce a considerable number of SANLCs that display typical electrophysiological characteristics of pacemaker cells in vitro.

**Conclusion:**

We provide an optimized and chemically defined protocol to generate SANLCs by combined modulation of WNT, RA, and FGF signaling pathways and metabolic selection by lactate enrichment and glucose starvation. This chemically defined method for generating SANLCs might provide a platform for disease modeling, drug discovery, predictive toxicology, and biological pacemaker construction.

**Supplementary Information:**

The online version contains supplementary material available at 10.1186/s13287-022-02834-y.

## Introduction

Human pluripotent stem cell-derived cardiomyocytes (hPSC-CMs) are increasingly used in cardiovascular research, including for disease modeling, safety testing, drug screening, cardiac development, and cellular therapy [1]. However, hPSC-CMs are not a specific cardiomyocyte subtype but a mixed cellular population comprising mostly ventricular and atrial cardiomyocytes and a small amount of sinoatrial node-like cells (SANLCs) [2]. This has greatly limited SANLC use for in vitro basic research and translational application into clinic. Generating SANLCs from hPSCs might allow to understand human sinoatrial node (SAN) diseases and to develop a biological pacemaker. Biological pacemakers constitute a promising alternative to electronic pacemakers, which have limitations including increased risk of infections, limited battery life, and a lack of hormonal responsiveness [3].

Multiple strategies have been developed to directly generate SANLCs in vitro. Keller and coworkers found that stage-specific activation of the bone morphogenic protein (BMP) and retinoic acid (RA) signaling pathways and inhibition of fibroblast growth factor (FGF) signaling at the cardiac mesoderm stage could enhance specification of SANLCs [4]. Our group recently established an approach to significantly enrich SANLCs by activating BMP signaling and simultaneous inhibiting RA and FGF pathways [5]. In addition, modulation of WNT/β-catenin (WNT) signaling using the exogenous WNT3a ligand or a glycogen synthase kinase 3 (GSK3) inhibitor also promotes pacemaker cell differentiation [6, 7]. Despite these progresses, little is known about the pathways involved in SANLC in vitro differentiation because of the undefined composition of the culture media, the relatively autonomous nature of in vitro cell differentiation, and the complexity of the secretome involved. Indeed, the protocols usually rely on the basal RPMI 1640 medium supplemented with B-27 (which is chemically undefined) and various cytokine including Activin A, bone morphogenic protein 4 (BMP4), vascular endothelial growth factor (VEGF), and basic FGF (bFGF). The B-27 supplement contains complex components derived from animals, which might have unpredictable influence for cardiomyocyte subtype specification [2, 8]. Moreover, the reproducibility of cytokines composition and the associated high cost are other limiting factors for studying SANLC in vitro differentiation [2]. Therefore, we sought to develop a novel, optimized, and low-cost cardiac differentiation protocol (without undefined medium components or cell factors) that would provide highly reproducible differentiation and allow further application in clinical research.

We established a reproducible and efficient method for differentiating SANLCs from hPSCs by synergistically modulating WNT, RA, and FGF signaling pathways with specific small molecules and using the chemically defined CDM3 medium. Up to 45% of SANLCs (defined by NKX2-5^−^cTnT^+^ cells) were produced with optimized concentration and treatment timing of the small molecules CHIR99021 (WNT signaling agonist), PD173074 (FGF signaling inhibitor), and BMS-189453 (RA signaling inhibitor). The enrichment reached approximately 50% of NKX2-5^−^cTnT^+^ cells when using lactate metabolism and glucose starvation. To our knowledge, this paper presents the first method to generate SANLCs from human pluripotent stem cell-derived lineage in vitro using fully defined chemicals.

## Materials and methods

### Human induced pluripotent stem cell culture

Human skin fibroblasts-derived induced pluripotent stem cells (HSF-iPSCs, provided by OSINGLAY BIO: HNF-P30-P11, China), urine-derived induced pluripotent stem cells (UiPSCs, provided by Cellapy: CA1002008, Beijing, China), and human embryonic pluripotent stem cells (hESCs-H9, provided by WiCell Institute Inc., Madison, WI, USA) were cultured as described previously [5]. Briefly, hiPSCs were routinely maintained with PSCeasy medium (Cellapy China). Cells were digested using 0.5 mM EDTA (Cellapy China) and resuspended with PSCeasy medium. 0.8–1.2 million cells were seeded per well in a 12-well plate coated with 1:200 growth factor-reduced Matrigel (9 μg/cm^2^). 2 µM thiazovivin (Selleck Chemicals) was added for the first 24 h after passage. hiPSCs were maintained by medium replacement daily in 3–4 days to reach 80%–90% confluence for differentiation. Cell lines were used between passages 20 and 50.

### Cardiomyocyte differentiation from hiPSCs

Cardiomyocyte differentiation was performed in CardioEasy medium (Cellapy China) by temporal modulation of the canonical WNT signaling pathway with GSK3 inhibitor and WNT inhibitor, as described in previous protocol [2]. CardioEasy medium, consisting of RPMI 1640 medium, 500 µg/ml O. sativa-derived recombinant human albumin (Sigma-Aldrich), and 213 µg/ml l-ascorbic acid 2-phosphate (Sigma-Aldrich), is abbreviated as CDM3 medium. Briefly, at day 0, 80–90% confluent hiPSCs were cultured in CDM3 medium with 6 µM CHIR99021 (SML1046, Sigma, USA) for 48 h. At day 2, medium was changed to CDM3 medium supplemented with 2 µM WNT-C59 (S7037, Selleck Chemicals) and continued to incubate for 48 h. Medium was refreshed on day 4 and every other day for CDM3 medium. The contracting cardiomyocytes can be noted from day 7.

### Optimization of concentration of small molecule chemicals

For day 0 to day 4, the cardiac mesoderm was inducted by bidirectional modulation of canonical WNT signaling pathway as above procedure. Different concentrations of CHIR99021 (1.25, 2.5, 5, 10 μM) and BMS-189453 (1.25, 2.5, 5, 10 μM) were added for day 4 to day 6, respectively. And different concentrations of PD173074 (0.48, 0.72, 0.96, 1.20 μM) were added for day 6 to day 8 (hiPSCs will dead and detach from the wall of plate if exposed to PD173074 from day4 to day6). Medium was refreshed with CDM3 after small molecule chemicals treatment for 48 h. The RT-PCR analysis was performed to evaluate the mRNA levels of SAN markers at day 24 to determine the optimal concentration of these small molecule chemicals.

### Optimization of administration timing of small molecule chemicals

For day 0 to day 4, the cardiac mesoderm was inducted by bidirectional modulation of canonical WNT signaling pathway as above procedure. Since day 4, optimal concentration of CHIR99021 (5 μM) and BMS-189453 (5 μM) was added at different time points of the differentiation process (day4–6, day5–7, day6–8, day 7–9), respectively. And optimal concentration of PD173074 (0.96 μM) was added at different time points (day5–7, day6–8, day 7–9). The RT-PCR analysis was performed to evaluate the mRNA levels of SAN markers at day 24 to determine the optimal administration timing of these small molecule chemicals.

### Combined treatment of three small molecule chemicals.

To investigate the synergistical effect, hiPSCs were treated with combinations of PD173074, CHIR99021, and BMS-189453 with the optimal concentration and administration timing, respectively. The markers of pacemaker cells were evaluated by analysis of RT-PCR, immunofluorescence, and flow cytometry at day 24.

### Purification of SANLCs by metabolic selection

Purification of SANLCs referenced a metabolic-selection method of cardiomyocytes as previous described [2, 9]. Briefly, SANLCs induced by three small molecule chemicals were performed in RPMI 1640 medium without d-glucose (11,879,020, Life Technologies) supplemented with 213 µg/ml l-ascorbic acid 2-phosphate (66,170–10-3, Sigma-Aldrich), 500 µg/ml O. sativa-derived recombinant human albumin (Sigma-Aldrich) for 2 days and then in RPMI 1640 medium without d-glucose supplemented with 213 µg/ml l-ascorbic acid 2-phosphate, 500 µg/ml O. sativa-derived recombinant human albumin, and 5 mM sodium dl-lactate (L4263, Sigma-Aldrich) for 3–4 days. Medium was changed to CDM3 medium for maintenance of SANLCs for 2 days. The markers of pacemaker cells were evaluated by analysis of RT-PCR and flow cytometry at day 32. Electrophysiological characteristics were analyzed using action potential (AP) recording at day 32.

### RNA isolation and quantitative real-time PCR

Total RNA was isolated from cells by TRIzol (Invitrogen, USA) and 1 μg RNA was reverse transcribed by Prime-Script™ reverse transcription kit (TaKaRa, Japan), following the manufacturer’s instructions. RNA was quantified with NANO drop 2000 (Thermo Fisher Scientific). The levels of relative gene expression were analyzed by quantitative reverse transcriptase PCR with TB Green™ Premix Ex Taq™ II (TaKaRa, Japan) using the 7500 Real-Time PCR System (Applied Biosystems, USA). The housekeeping gene GAPDH was used for internal normalization and the relative quantification of gene expression was calculated according to the ΔCT method. The RT-PCR primers are listed in Additional file 2: Table S1.

### Flow cytometry

Cells were dissociated into single cells using CardioEasy cardiomyocyte dissociation buffer (Cellapy, China), fixed with chilled 4% formaldehyde (Beyotime, China) for 15–20 min at room temperature; permeabilized with ice-cold 0.2% Triton X-100 (Invitrogen, USA) for 15–20 min at room temperature, blocked with 0.5% bovine serum albumin (Sigma-Aldrich, USA) for 10 min at room temperature, and then incubated with the following primary antibodies for 1 h: anti-cTNT antibody (ThermoFisher Scientific, 1:1000), anti-NKX2-5 antibody (abcam, 1:200), anti-SHOX2 antibody (abcam, 1:500). And fluorescence-conjugated secondary antibodies for 30 min at room temperature: Goat Anti-Rabbit IgG Alexa Fluor 488 (ab150077, abcam, USA), Goat Anti-Mouse IgG Alexa Fluor 488 (ab150113, abcam, USA), Goat Anti-Rabbit IgG Alexa Fluor 647 (ab150079, abcam, USA), and Goat Anti-Mouse IgG Alexa Fluor 647 (ab150115, abcam, USA). Detailed antibody information is described in the Additional file 2: Table S2. Cells were washed with PBS for three times. Finally, cells were analyzed using a flow cytometry machine (BD Accuri C6, BD Bioscience, USA) according to the manufacturer’s protocol. Data were analyzed with FlowJo V10 software.

### Immunofluorescence staining

Cells were dissociated into single cells using CardioEasy cardiomyocyte dissociation buffer (Cellapy, China), fixed with chilled 4% formaldehyde (Beyotime, China) for 15–20 min at room temperature; permeabilized with ice-cold 0.5%Triton X-100 (Invitrogen, USA) for 15–20 min at room temperature, blocked with 5% bovine serum albumin (Sigma-Aldrich, USA) for 30 min at room temperature, and then incubated with the following primary antibodies: anti-α-Actinin antibody (abcam, 1:100), anti-cTNT antibody (ThermoFisher Scientific, 1:500), anti-NKX2-5 antibody (abcam, 1:200), anti-SHOX2 antibody (abcam, 1:250) overnight at 4 °C; and followed by incubation with the corresponding species-specific fluorescence-conjugated secondary antibodies: Goat Anti-Rabbit IgG Alexa Fluor 488 (ab150077, abcam, USA), Goat Anti-Mouse IgG Alexa Fluor 488 (ab150113, abcam, USA), Goat Anti-Rabbit IgG Alexa Fluor 647 (ab150079, abcam, USA), and Goat Anti-Mouse IgG Alexa Fluor 647 (ab150115, abcam, USA) for 1 h at room temperature. Detailed antibody information is described in the Additional file 2: Table S2. Wash with PBS three times before each step. Nuclei were stained with DAPI (4083, Cell Signaling Technology, USA) for 15 min at room temperature. Fluorescence images were captured by Leica DMI 4000B fluorescence microscope and Leica TCS SP5 MP confocal laser scanning microscope (Leica, Germany). Data were analyzed with Image J software.

### Patch clamp

32-day cells were used to record cellular action potentials (APs). Cells were dissociated using CardioEasy cardiomyocyte dissociation buffer I (Cellapy, China) for 30 min followed by CardioEasy® cardiomyocyte dissociation buffer II (Cellapy, China) for 20 min, plated as single cells (1–2 × 10^4^ cells) into a 3.5-cm dish containing a 6-mm round cover glasses (VWR Collection) coated with 1:200 growth factor-reduced Matrigel (9 μg/cm2) in CDM3 medium with 2 µM thiazovivin and incubated for 3 days. Cultures were used for patch clamp recordings 4–10 days following plating.

The adherent cells on the coverslip were transfer to the recording chamber mounted onto the stage of an inverted microscope (Olympus, Japan) for whole-cell patch clamp. Cell cultures were perfused with warm (35–37 °C) bath solution consisting of 137 mM NaC1, 4 mM KCl, 1 mM MgCl2 6H2O, 1.8 mM CaCl2 2H2O, 10 mM HEPES, and 10 mM D-Glucose; pH was adjusted to 7.35 with NaOH. Pipette electrodes were made from borosilicate glass electrodes (outer diameter 1 mm) (7–000-0650-LHC, Drummond, USA) using a micropipette Puller (PC100, NARISHIGE, Japan) and had resistances of 1.5–3 MΩ. Pipette solution consisted of 20 mM KCl, 110 mM K-Aspartate, 5 mM Mg2-ATP, 0.1 mM GTP, 5 mM EGTA, 10 mM HEPES, and 5 mM Na-phosphocreatine; pH was adjusted to 7.2 by KOH, and the osmolality to 290 ± 3 mOsm.

APs were measured by using the current clamp mode of MultiClamp 700B amplifier (AXON Instruments, USA). Voltages were recorded with 5 kHz sampling rate (DIGIDATA1440A, Narishige Japan) and analyzed with Clampfit 10.4 software. Ventricular-like cells had typical APs with a negative maximum diastolic membrane potential (< − 50 mV), a rapid AP upstroke, a long plateau phase, AP amplitude > 90 mV, and AP duration at 90% repolarization/AP duration at 50% repolarization (APD90/APD50) < 1.4. For atrial-like cells, the criteria were an absence of a prominent plateau phase, a negative diastolic membrane potential (< − 50 mV), and APD90/APD50 > 1.7. SANLCs showed a more positive MDP, slower AP upstroke, prominent phase 4 depolarization, and moderate APD90/APD50 (1.4–1.7).

### Data analysis and statistics

Data were plotted and analyzed using GraphPad Prism version 8. All experimental data are analyzed by means ± standard errors of the means (S.E.M.). Statistical significance was evaluated using unpaired t-test for two groups and using one-way ANOVA test for statistical differences of multiple groups. Significant differences were considered when *P* < 0.05.

## Results

### Identification and characterization of SANLCs differentiated in CDM3 medium

**Fig. 1:**
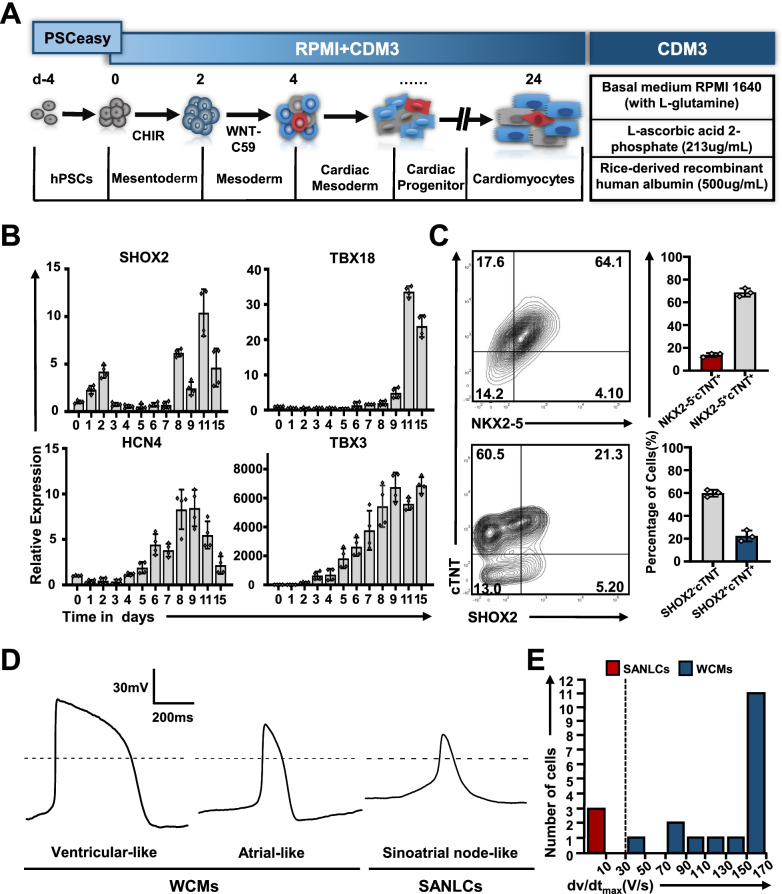
Identification and characterization of SANLCs derived from hPSCs in a CDM3-based differentiation method. **a** Scheme of the CDM3-based differentiation protocol used for cardiomyocyte differentiation from hPSCs. **b** Relative expression of SHOX2, TBX18, HCN4, and TBX3 mRNA by using chemically defined cardiac differentiation protocol. Values represent expression levels relative to the housekeeping gene GAPDH (n = 4). **c** Representative flow cytometric analyses of the proportion of NKX2-5^−^cTNT^+^ and SHOX2^+^cTNT^+^ cells in CDM3-based differentiation method at day 24 (n = 3). **d** Representative action potential recordings using whole-cell patch of three cardiomyocyte subtypes produced from day 32 induced cells (total = 20 cells). **e** Histogram plot showing the distribution of the maximum upstroke velocities (dV/dt_max_) recorded in 20 cardiomyocytes. SANLCs displayed slow maximum upstroke velocities (< 30 V/s), while WCMs displaying fast upstroke velocities (> 30 V/s). SANLCs, sinoatrial node-like cells; hPSCs, human pluripotent stem cells; WCMs, working cardiomyocytes; CHIR, CHIR99021, WNT signaling agonist; WNT-C59, WNT signaling inhibitor

To determine the proportion of SANLCs in the cardiomyocyte population differentiated in CDM3 medium (Fig. 1a), we performed the experiments with three different hPSC lines (HSF-iPSCs, human skin fibroblasts-derived induced pluripotent stem cells; UiPSCs, urine-derived induced pluripotent stem cells; hESCs-H9, human embryonic pluripotent stem cells) following the previously described protocol [2]. We observed 80–90% contractile cardiomyocytes at day 7 as shown in Additional file 3: Video S1 and Additional file 1: Fig. S1. We also descripted the relevant gene expression profile during cardiomyocyte differentiation, which is consistent with the previous report (Additional file 1: Fig. S2). The expression of transcription factors involved in the development of the SAN, including T-box transcription factor 18 (TBX18), short stature homeobox protein 2 (SHOX2), and TBX3, was upregulated on day 3–8 of differentiation, suggesting that pacemaker cells were generated under these conditions (Fig. 1b). Cells were analyzed by flow cytometry at day 24 of differentiation to identify the level of SANLCs. The proportion of homeobox transcription factor NKX2-5-negative and cardiac troponin T (cTNT)-positive (NKX2-5^−^/cTNT^+^) and SHOX2^+^/cTNT^+^ cells (Both of cells being considered as SANLCs) were 13.7% ± 3% and 22.4% ± 3%, respectively (Fig. 1c). In addition, we recorded action potentials (APs) from 20 cells at day 32 of differentiation using whole-cell patch clamp. Electrophysiological analyses revealed that 15% of cardiomyocytes induced with the CDM3-based differentiation method had typical pacemaker APs with fast spontaneous firing rates, slow maximum upstroke velocities (< 30 V/s), small AP amplitudes, and short AP durations. In contrast, 85% of cardiomyocytes displayed fast upstroke velocities (> 30 V/s) and long AP durations (AP duration at 50% of repolarization > 100 ms) typical from working cardiomyocytes (Fig. 1d–e and Additional file 2: Table S3).

### Optimization of concentration and administration timing of small molecule chemicals to improve SANLCs differentiation

**Fig. 2:**
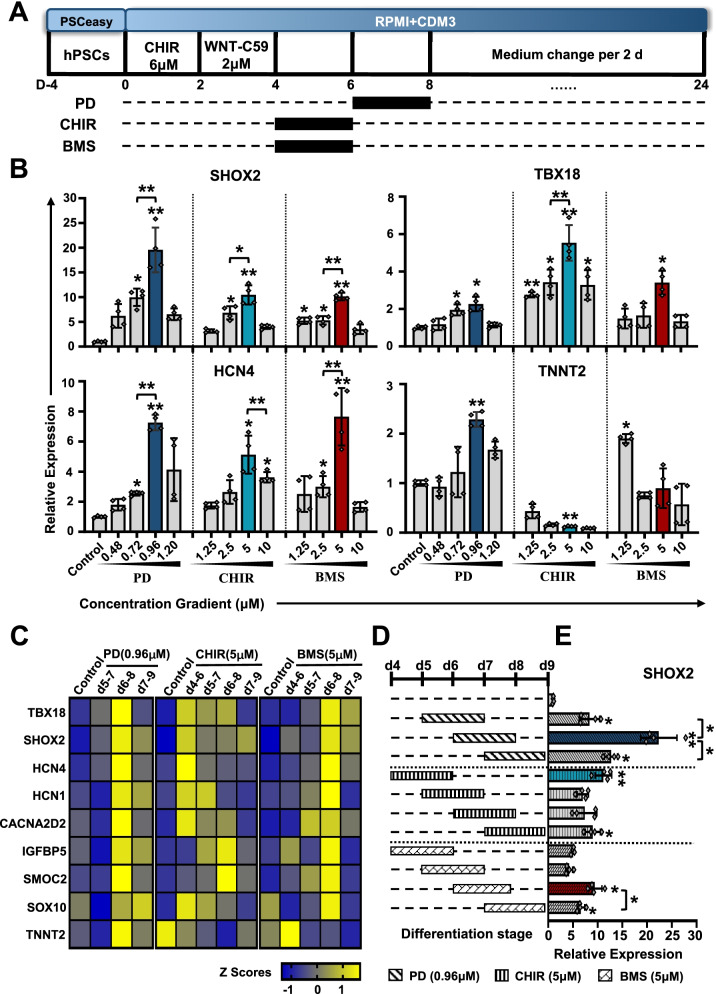
Optimization of the concentration and timing of three small molecule chemicals to enrich SANLCs differentiation. **a** Scheme of the protocol to test the optimal concentration of three small molecule chemicals. **b** Relative expression of SHOX2, TBX18, HCN4, and TNNT2 mRNA at day 24 when treated with PD, CHIR, and BMS. Values represent expression relative to the housekeeping gene GAPDH. One-way ANOVA followed by Bonferroni’s post hoc test: *P < 0.05, **P < 0.01 versus untreated control or indicated sample (n = 4). **c** Heatmap shows expression changes of genes related to SAN in induced cells at days 24 following treatment with 0.96 μM PD, 5 μM CHIR, and 5 μM BMS, respectively. **d** Schematic diagram indicating the timing of PD, CHIR, and BMS. **e** RT-PCR analysis of the expression of pacemaker transcription factor SHOX2. Values represent expression relative to the housekeeping gene GAPDH. One-way ANOVA followed by Bonferroni’s post hoc test: *P < 0.05, **P < 0.01 versus untreated control or indicated sample (n = 4). All error bars represent the SD of four independent experiments. CHIR, CHIR99021; BMS, BMS-189453; PD, PD173074

Multiple signaling pathways contribute to the enrichment of SANLCs in vitro, including the nodal, BMP, WNT, FGF, and RA signaling [4–6]. To improve the differentiation efficiency of SANLCs in CDM3 medium, we applied three small molecule chemicals: PD173074, CHIR99021, and BMS-189453 at the cardiovascular specification stage (Fig. 2a). To determine the optimal concentration of these chemicals, we assessed the expression level of pacemaker genes at day 24 of differentiation by RT-PCR after treatment with different concentrations of the chemicals. All concentrations of CHIR99021, BMS-189453, and PD173074 promoted the expression of SHOX2, TBX18, and potassium/sodium hyperpolarization-activated cyclic nucleotide-gated channel 4 (HCN4) at day 4–8. The expression of SAN markers was significantly increased by 0.96 µM PD173074, 5 µM CHIR99021, and 5 µM BMS-189453. However, these concentrations induced different expression of the pan-cardiomyocyte marker cardiac troponin T, TNNT2 (Fig. 2b). Next, we used these concentrations to identify the optimal time window for the administration of the small molecules (Fig. 2c). In brief, we added 0.96 µM PD173074, 5 µM CHIR99021, and 5 µM BMS-189453 for 48 h starting between day 4 and day 9 of hPSCs differentiation and measured the expression levels of SAN markers. CHIR99021 significantly improved the expression of SAN-related genes when administrated from day 4 to 6. The optimal timing of PD173074 and BMS-189453 treatment was from day 6 to 8 (Fig. 2c–e).

Altogether, these findings demonstrate that all three small molecule chemicals play an important role in the specification of cardiac mesoderm to a SAN fate. Moreover, this specification step depends on the concentration and the treatment time window.

### Synergetic modulation of FGF, WNT, and RA signaling pathways enhances SANLCs enrichment

The specification of the cardiovascular mesoderm to a subtype progenitor fate is controlled by the combined activation of several pathways including the nodal, WNT, BMP, FGF, RA, and VEGF signaling [8, 10–12]. The spatiotemporal activation of these pathways is highly coordinated to progressively direct hPSCs into specific cell lineages through a series of fate decisions. Therefore, we investigated the effects of PD173074, CHIR99021, and BMS-189453 synergetic treatment on the differentiation of hPSCs to SANLCs.

**Fig. 3:**
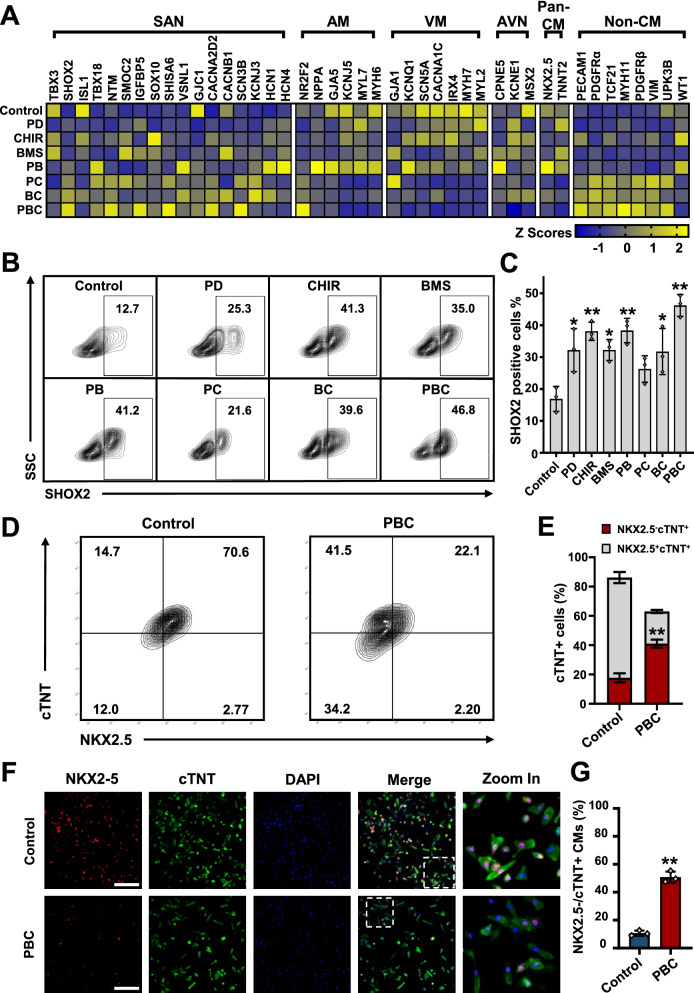
Combination of three small molecule chemicals synergistically promoted the differentiation of SANLCs. **a** Relative expression of SAN, atrial cardiomyocyte, ventricular cardiomyocyte, atrioventricular node, pan-cardiomyocyte, and non-cardiomyocyte gene via qPCR. **b**, **c** Flow cytometry of seven different groups treated with combinations of PD, CHIR, and BMS at day 24. *P ≤ 0.05, **P ≤ 0.01 compared to control group as analyzed by Student’s t-test (n = 3). **d**, **e** Representative flow cytometric analyses of the proportion of NKX2-5^−^/cTNT^+^ cells for control and PBC group at day 24. **P ≤ 0.01 by Student’s t test (n = 3). **f**, **g** Immunofluorescent staining of NKX2-5 and cTNT in PBC group (Bar = 250 μm). **P ≤ 0.01 by Student’s t test (n = 3). All error bars represent the SD of three independent experiments. PD, PD173074; CHIR, CHIR99021; BMS, BMS-189453; PB, PD173074 + BMS-189453; PC, PD173074 + CHIR99021; BC, BMS-189453 + CHIR99021; PBC, PD173074 + BMS-189453 + CHIR99021

The expression of genes characteristic of SAN, atrial cardiomyocytes, ventricular cardiomyocytes, atrioventricular node, pan-cardiomyocytes, and non-cardiomyocyte cells (including endothelial cells, vascular smooth muscle cells, fibroblasts, and epicardial cells) was analyzed by RT-PCR at differentiation day 24. PD173074, CHIR99021, and BMS-189453 were combined as follows: PD173074 + BMS-189453 (abbreviated PB), PD173074 + CHIR99021 (abbreviated PC), BMS-189453 + CHIR99021 (abbreviated BC), and PD173074 + BMS-189453 + CHIR99021 (abbreviated PBC). All combinations significantly stimulated SAN-related gene expression (Fig. 3a). Interestingly, the levels of SAN-related genes, including SHOX2, insulin like growth factor binding protein 5 (IGFBP5), shisa family member 6 (SHISA6), and sodium voltage-gated channel beta subunit 3 (SCN3B) [13, 14], were especially elevated in the PBC condition, whereas the expression of atrial, ventricular, and atrioventricular node markers was downregulated in three hPSC lines (Fig. 3a and Additional file 1: Fig. S4A). The proportion of SHOX2^+^ cells, which were considered pacemaker cells [15], was significantly increased in the PBC group, further supporting the RT-PCR results (Fig. 3b–c).

Given that SANLCs are defined as NKX2-5^−^/cTNT^+^ or SHOX2^+^/cTNT^+^ [4, 6], we further evaluated the level of NKX2-5 and SHOX2 in cTNT^+^ cells using flow cytometry and immunofluorescence analyses. Flow cytometry data indicated that the addition of PBC substantially increased the proportion of SANLCs generated to 41.0% ± 3% (HSF-iPS), 45.0% ± 3% (UiPS), and 35.0% ± 2% (H9) of NKX2-5^−^/cTNT^+^ cells and to 44.6% ± 3% (HSF-iPS) of SHOX2^+^/cTNT^+^ cells compared to the levels obtained with the CDM3 induction protocol (17.7% ± 3% (HSF-iPS), 23.0 ± 2% (UiPS), and 10.1 ± 2% (H9) of NKX2-5^−^/cTNT^+^ cells and 24.5 ± 2% (HSF-iPS) of SHOX2^+^/cTNT^+^ cells) (Fig. 3c–e, Additional file 1: Fig. S3a–b, and Fig. S4b–d). These data were consistent with the immunofluorescence staining results (Fig. 3f–3g and Additional file 1: Fig. S3c–d). In conclusion, the synergetic modulation of WNT, RA, and FGF signaling pathways promoted the differentiation of SANLCs (Additional file 4: Video S2).

### Metabolic purification of SANLCs generated by chemically defined differentiation

**Fig. 4:**
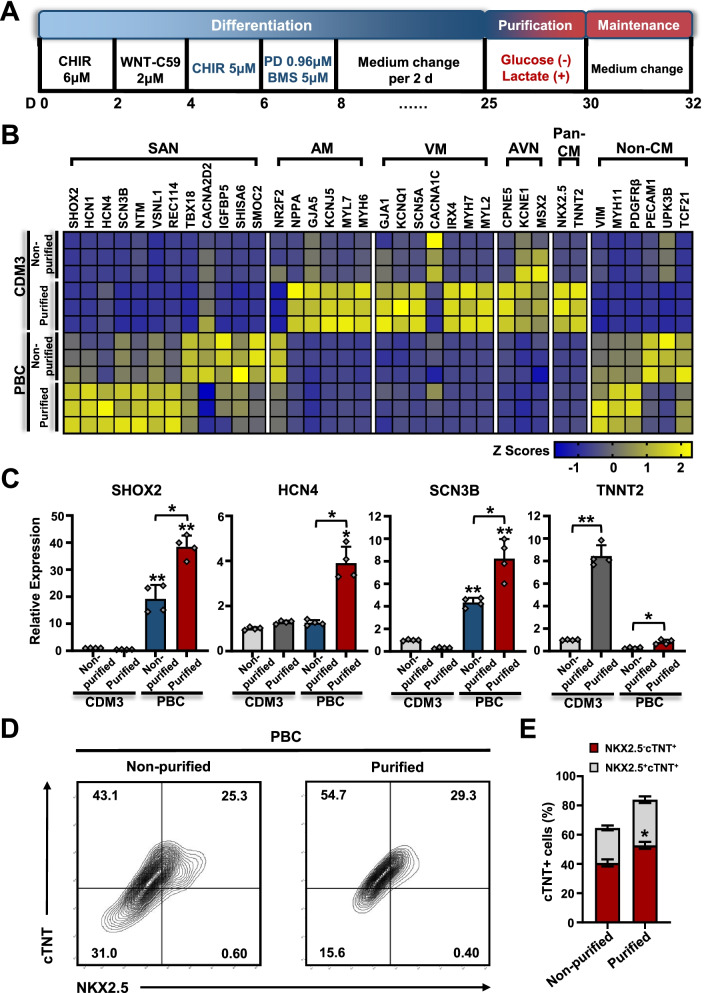
Enrichment of SANLCs with metabolic selection. **a** Scheme of SANLCs metabolic selection. **b** Heatmap shows expression changes of genes related to SAN, atrial cardiomyocyte, ventricular cardiomyocyte, atrioventricular node, pan-cardiomyocyte, and non-CMs cells at day 32 via qPCR. **c** Relative expression of SHOX2, HCN4, SCN3B and TNNT2 mRNA. Values represent expression relative to the housekeeping gene GAPDH. One-way ANOVA followed by Bonferroni’s post hoc test: *P < 0.05, **P < 0.01 versus untreated control or indicated sample (n = 4). **d**, **e** Representative flow cytometric analyses of PBC group following metabolic selection at day 32. *P < 0.05 by Student’s t test (n = 3). All error bars represent the SD of three or four independent experiments

The PBC protocol produced the best induction efficiency compared with that obtained with other combinations. However, it also increased the expression of marker genes for endothelial cells (platelet endothelial cell adhesion molecule, PECAM1), fibroblasts (platelet-derived growth factor receptor alpha, PDGFRa and transcription factor 21, TCF21), vascular smooth muscle cells (myosin 11, MYH11), PDGFRb, vimentin (VIM), and epicardial cells (uroplakin-3b, UPK3B) (Fig. 3a). To increase the purity of SANLCs, we treated the mixed cells with a metabolic selection medium to remove non-cardiomyocyte cells as described previously [9]. We cultured the cells with CDM3 medium without D-glucose for 2 days and then with metabolic selection medium containing 5 mM sodium DL-lactate and no D-glucose for 3–4 days (Fig. 4a). Compared with PBC group without metabolic selection, the expression of SAN-related genes and the pan-cardiomyocyte marker TNNT2 in the PBC group after metabolic selection was significantly stimulated, whereas the non-cardiomyocyte marker levels were partly decreased as assessed by RT-PCR (Fig. 4b–c). We then performed flow cytometry assays to determine the proportion of SANLCs after metabolic selection. The proportion of SANLCs defined by NKX2-5^−^/cTNT^+^ reached 52.7% ± 3% after metabolic selection against 40.7% ± 3% without metabolic selection. Similarly, the metabolic selection yielded 52.2% ± 3% SANLCs defined by SHOX2^+^/cTNT^+^, whereas 39.8% ± 3% were recovered without metabolic selection (Fig. 4d–e and Additional file 1: Fig. S3e–f). These findings indicated that the purification based on lactate metabolism and glucose starvation further increased the proportion of SANLCs (Additional file 5: Video S3).

Next, we compared current protocol with two available protocols of generating SANLCs in vitro provided by Ren et al. (CHIR-protocol) [6] and Liu et al. (BPM-protocol) [5], side by side. The gene expression and differentiation efficiency of SANLCs derived from hESCs-H9 in vitro were analyzed by RT-PCR and flow cytometry assays at differentiation day 24 of three different methods (including current protocol). As indicated in Additional file 1: Fig. S5a, current protocol (PBC group after metabolic selection) significantly stimulated SAN-related gene expression compared with the other two protocols. Flow cytometry data indicated that the current protocol increased the proportion of SANLCs generated to 45.9% ± 3% of NKX2-5^−^/cTNT^+^ cells compared to the levels obtained with the CHIR-protocol proposed by Ren et al. (33.6% ± 4% of NKX2-5^−^/cTNT^+^). Nevertheless, the proportion of SANLCs generated with the BPM-protocol by Liu et al. was similar to that obtained in current protocol (Additional file 1: Fig. S5b–c).

### SANLCs generated under chemically defined conditions display the typical electrophysiological characteristics of pacemaker cells

**Fig. 5:**
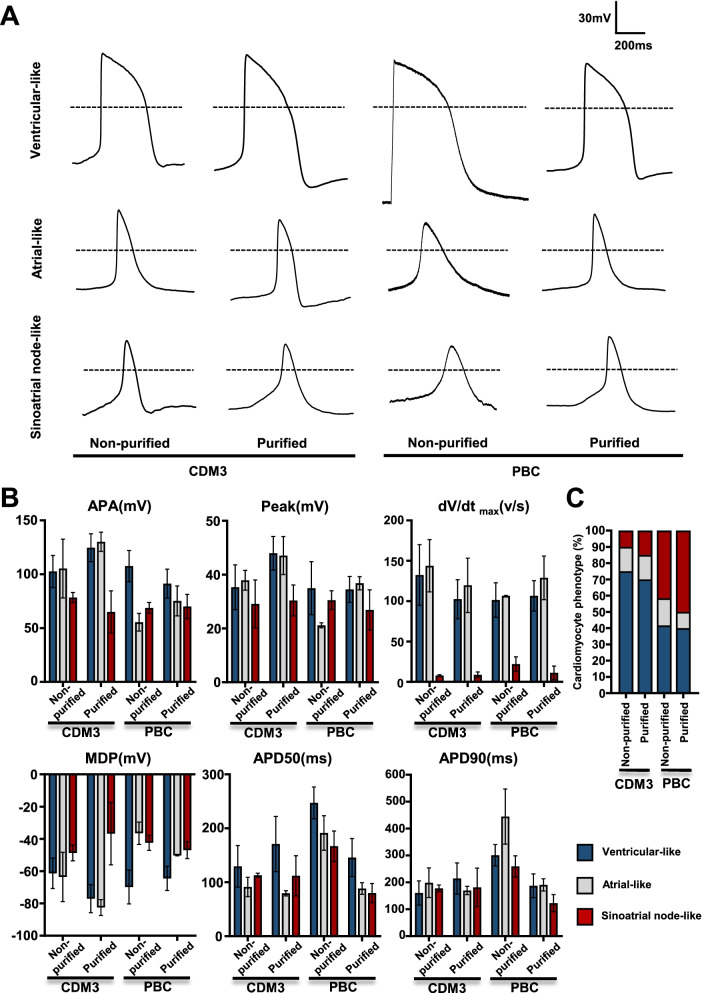
Electrophysiological characterization of SANLCs generated under chemically defined conditions.** a** Representative action potential recording using whole-cell patch from non-purified CDM3 group, purified CDM3 group, non-purified PBC group, and purified PBC group. **b** Patch clamp recordings of induced cells from non-purified CDM3 group (20 cells), purified CDM3 group (20 cells), non-purified PBC group (12 cells), and purified PBC group (20 cells), demonstrating MDP, peak voltage, APA, APD90, APD50, and dV/dt_max_ (maximal rate of depolarization). **c** Bar graph indicates proportions of cardiomyocyte subtypes at day 32. MDP, maximum diastolic potential; APA, action potential amplitude; APD, action potential duration at different levels of repolarization (90% and 50%)

To evaluate the electrophysiological characteristics of the SANLCs generated with the protocol described above, we analyzed the AP amplitude, peak voltage, maximum diastolic potential, maximal rate of depolarization (dV/dtmax), and ratio of AP duration at 90% repolarization (APD90) to APD50 of induced cells by whole-cell patch clamp at differentiation day 32. The results revealed that the cells induced using the PBC protocol with/without purification, or CDM3 differentiation protocol with/without purification contained ventricle-like, atrial-like, and nodal-like cells (Fig. 5a). Typical SAN-like AP with fast spontaneous firing rates, slow maximum upstroke velocities (< 30 V/s), small AP amplitudes, and short AP durations were observed in 50% (10/20) of cells induced by PBC protocol with purification and 42% (5/12) of cells induced by PBC protocol without purification. The difference between the two rates was not statistically significant according to Pearson Chi-Square Tests (χ^2^ = 0.209, *P* > 0.05). In contrast, the majority (75%) of cells induced by CDM3 method with/without purification displayed fast upstroke velocities (> 30 V/s) and long AP durations (AP duration at 50% of repolarization > 100 ms) typical of ventricular cardiomyocytes (Fig. 5b–c and Additional file 2: Table S3). And there is no difference in electrophysiological characteristics of cells induced by CDM3 method between purified group and non-purified group.

In addition, the cells induced using the current protocol displayed less atrial-like AP with negative diastolic membrane potential (< − 50 mV), absence of a prominent plateau phase, and APD90/APD50 ratio > 1.7 than using the BPM-protocol proposed by Liu et al. (Additional file 1: Fig. S6 and Additional file 2: Table S3). And compared to the CHIR-protocol proposed by Ren et al., the cells induced using the current protocol displayed more SANLCs that displayed SAN-like AP with slow AP upstroke, prominent phase 4 depolarization, and short AP durations (Additional file 1: Fig. S6 and Additional file 2: Table S3).

Therefore, the electrophysiological data were consistent with the flow cytometry and RT-PCR findings. They demonstrated that cells differentiated with the proposed protocol produce a considerable number of SANLCs that display typical electrophysiological characteristics of pacemaker cells in vitro.

## Discussion

This study proposes a method for the efficient and reproducible generation of SANLCs that display molecular and electrophysiological properties of native SAN cardiomyocytes. Based on the chemically defined platform for cardiac subtype specification of hPSCs, we showed that the synergistic modulation of WNT, RA, and FGF signaling pathways using three small molecules promoted the differentiation of hPSCs to SANLCs. In addition, the purification based on lactate metabolism and glucose starvation further increased the proportion of SANLCs. Therefore, we provide a novel, optimized, and low-cost strategy for generating unlimited numbers of SANLCs from any hPSCs line.

The embryonic SAN development experiences several spatial–temporal stages from primitive streak to cardiac mesoderm and then to second heart field, and the posterior second heart field progenitors recruit the Tbx18^+^ mesenchymal precursors to form SAN primordia [15]. The differentiation of SANLCs derived from hPSCs in vitro may be determined by critical signaling pathways that are required in the development of embryonic SAN in vivo. Jie Ren et al. found that WNT signaling modulation with exogenous WNT3a ligand or GSK3 inhibitor promoted pacemaker cells differentiation by coordinating gene regulatory networks to direct mesoderm cell fate decisions [6]. Protze et al. showed that modulating the BMP and FGF signaling pathways enables highly efficient SAN-like cell induction [4]. Recently, our group uncovered that inhibiting RA signaling contributes significantly to the differentiation of SANLCs [5]. The specification of cardiac mesoderm cell fate to SAN cell fate may be regulated by combinatorial signaling of a sequence of pathways that are required in a highly coordinated spatiotemporal manner. Accordingly, the present study shows that the combined modulation of WNT, RA, and FGF signaling pathways, compared to the effects of single signaling pathway modulation, induced the highest increase of SAN-related gene expression and a decrease in the levels of atrioventricular node, ventricular, and atrial markers (except nuclear receptor subfamily 2 group F member 2 or NR2F2, a transcription factor expressed at high levels in the SANLCs and fetal SAN) [4]. Moreover, the proportion of SHOX2^+^ cells, which were considered pacemaker cells [16], was significantly increased when WNT, RA, and FGF signaling pathways were synergistically modulated. Therefore, the temporal and spatial regulation pattern of multiple pathways that simulate the embryonic SAN development in vivo may be a promising way for the generation of SANLCs in vitro.

But, treating hPSCs with the WNT signaling activator CHIR99021 during the cardiac mesodermal stage blocks the specification of the cardiovascular mesoderm to a cardiomyocyte fate and promotes the differentiation of human pluripotent stem cells to endothelial progenitor cells or proepicardial cells [12, 17, 18]. In line with these observations, the combined modulation of WNT, RA, and FGF signaling pathways extremely reduced the pan-cardiomyocyte yield and increased the proportion of non-cardiomyocyte cells as assessed by RT-PCR and flow cytometry, which limited deeply the purification and use of SANLCs.

To remove non-cardiomyocyte cells in induced SANLCs, we treated the mixed cells with a metabolic selection medium. Tohyama et al. showed marked biochemical differences in glucose and lactate metabolisms between cardiomyocytes and non-cardiomyocyte cells, including undifferentiated cells. Non-cardiomyocytes mainly dependent on glycolysis might not be able to survive under glucose-depleted and lactate-abundant conditions, whereas cardiomyocytes would survive by using lactate as an alternative energy source [9]. In current study, the proportion of non-cardiomyocyte cells was significantly reduced after metabolic selection. The expression of non-cardiomyocyte marker genes was downregulated, except for vascular smooth muscle marker genes, possibly because immature cardiomyocytes also express smooth muscle actin [9]. Therefore, SANLCs, as one of the pan-cardiomyocyte pool, may also survive under glucose-depleted and lactate-abundant conditions. Indeed, earlier studies have observed that the Isolated, native sinoatrial node cells exhibited a remarkable resistance and resilience to prolonged hypoxia followed by reoxygenation [19]. Gu et al. showed TBX18-induced pacemaker myocytes (iPMs) exhibited negligible cell death after 2 days of near anoxia and/or inhibition of glycolysis, demonstrating that the iPMs displayed low metabolic demand and high resistance to hypoxic stress and glucose starvation similar to the native pacemaker tissue [20]. Although the biochemical difference in metabolism between SANLCs and chamber-like cardiomyocytes derived from hPSCs is not clear, these findings also support our results that SANLCs displayed distinct metabolic flow from non-cardiomyocyte cells, which enabled further purification of SANLCs derived from hPSCs.

Interestingly, we found a downregulation of some SAN-related genes after metabolic selection for cardiomyocyte purification. This is in line with single-cell analysis of hPSCs-CMs in glucose-depleted and lactate-enriched culture conditions [21]. In fact, these downregulated genes expressed not only in the SAN cells, but also in the non-cardiomyocyte cells. For example, TBX18 and SHISA6 are the marker gene of epicardial cells and cerebellar Purkinje cells, respectively [18, 22]. In addition, there may be some SANLCs that are not fully differentiated or immature SANLCs that also expressed mRNA of SAN-related gene during differentiation. Due to the lack of relevant protein expression, these cells cannot utilize lactate and tolerate glucose starvation.

Our electrophysiological data suggested the metabolic selection might not change cardiomyocyte subtype. In fact, the enrichment differentiation of SANLCs using the PBC protocol depended on the modulation of signaling pathways to regulate cardiac subtype myocyte lineage specification. But the activation of WNT signaling during the cardiac mesodermal stage will delay the differentiation progress of pan-cardiomyocyte fate, increasing the heterogeneity of induced cells derived from second heart field progenitors [6, 23]. We found the action potential of cells induced using the PBC protocol without purification need to be evoked by electric currents, while the cells induced using the PBC protocol with purification usually displayed spontaneous action potential, which might be because of the significant up-regulation of the expression of SAN-related genes especially in ion channel in the PBC group after metabolic selection, such as HCN4, HCN1, and SCN3B. Our results are also in line with previous observations. Tohyama et al. showed that purified hPSC-derived cardiomyocytes will show high proliferative capacity, physiologically relevant action-potential configurations, and drug responses [9]. Saito Y et al. showed that purified HCN4-overexpressing mESC-CMs (under serum/glucose-free and lactate-supplemented conditions) showed significantly larger If currents and more rapid spontaneous beating than did non-overexpressing mESC-CMs [24].

Taken together, metabolic selection might be a promising approach not requiring fluorescence-activated cell sorting for further purifying SANLCs inducted by modulation of signaling pathways. But the current metabolic selection method might not change cardiomyocyte subtype according to our and others’ electrophysiological data [9, 25]. There are, until now, no effective purification methods for distinguishing SANLCs from chamber-like cardiomyocytes derived from hPSCs, even though the degree of oxidative phosphorylation and overall global metabolic demand are significantly lower in pacemaker myocytes compared with those in the working cardiomyocytes [20]. Therefore, the effects of metabolic manipulation of SANLCs need to be further investigated for SANLCs generation in vitro.

Previous strategies for generating SANLCs focused on either transgene-dependent or cytokine-dependent approaches. The transgene-dependent approaches, including in vitro reprogramming of pluripotent stem cells with TBX3 or SHOX2 [26, 27], and in vivo reprogramming of chamber cardiomyocytes with TBX18 [28, 29], relied on genetic modifications of the target cells, which might compromise the safety of the derivative population for future clinical applications. The cytokine-dependent approaches for generating SANLCs required the combined modulation of various signaling pathways using various cytokines including activin A, BMP4, VEGF, and bFGF. The use of cytokines poses problems of reproducibility and is expensive. In addition, BMP4 also induces activin A/nodal signaling in mesodermal populations, which might have unpredictable influence on the induction of SANLCs [4, 10]. Here, we used CDM3 medium and small molecular components to establish a reproducible and efficient differentiation method under chemically defined conditions without any exogenous cytokines and cross talk between various signaling pathways. The proportion of SANLCs generated with the proposed protocol was similar to that obtained in previous investigations [4–6]. However, the cells induced using the current protocol contained less atrial-like cells than the induction protocol proposed by Liu et al. [5] and more SANLCs than the induction protocol proposed by Ren et al. [6]. While compared to the induction protocol proposed by Protze et al. [4], the current chemically defined protocol (without unknown medium components or cell factors) might serve as a simpler, lower-cost, and more reproducible strategy for SANLCs generation. Based on this protocol, we can create a hESCs/iPSCs fluorescence-reporter system (such as NKX2-5^GFP^ reporter [4, 30]) to differentiate into SANLCs in vitro reproducibly and produce a considerable number of SANLCs by fluorescence-activated cell sorting for disease modeling, drug discovery, predictive toxicology. And then, we can investigate how to promote the mature nature of SANLCs for biological pacemaker construction, such as some already available strategies for cardiomyocytes, electrical stimulation, and so on [31].

Although we provide a small molecule-optimized and metabolic selection-based method for generating SANLCs, the differentiation efficiency of SANLCs in vitro requires further improvements for developing clinically compliant biological pacemakers. Given that ventricular, atrial, and SAN-like cardiomyocytes are derived from different progenitor cells emerging as early as cardiovascular mesoderm stage [11], it is probable that the SAN progenitor fate has been determined at a differentiation stage preceding the cardiovascular mesoderm. On the contrary, in the present work, all treatments with signal pathway modulators to enhance the pacemaker characteristics occur at a differentiation stage after the induction of cardiovascular mesoderm. It might be too late for the specification of the cardiovascular mesoderm to a SAN fate. Therefore, further work is needed to determine the time window for inducing SAN progenitors at the differentiation stage before the cardiovascular mesoderm to allow the direct differentiation of SANLCs from hPSCs.

## Conclusion

**Fig. 6:**
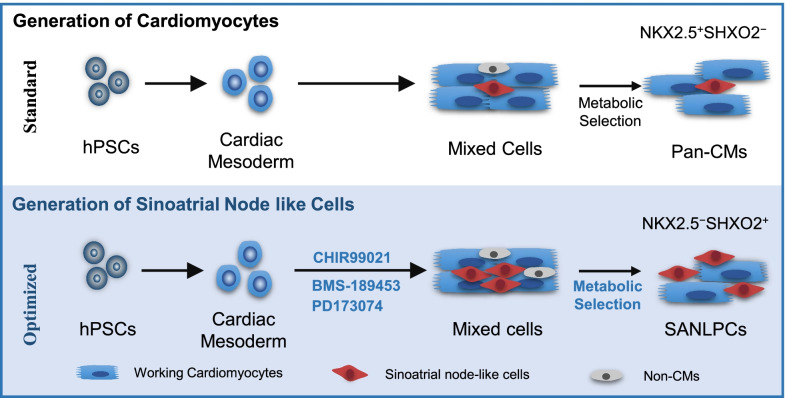
Graphic abstract. Comparison of strategy used for the differentiation and purification of pan-cardiomyocytes and SANLCs under chemically defined conditions

In summary, we provide an optimized and chemically defined protocol to generate SANLCs by combined modulation of WNT, RA, and FGF signaling pathways and metabolic selection by lactate enrichment and glucose starvation (Fig. 6). Thanks to this method, it will be possible to model diseases of SAN dysfunction using patient-derived iPSCs and provide the opportunity to design human SAN-based platforms for drug discovery and predictive toxicology.

## Supplementary Information

**Additional file 1: Fig. S1**. Characterization of the hPSC-derived cardiomyocytes. (A) Morphological contrast image of different hPSC lines and hPSC-derived cardiomyocytes during chemically defined differentiation. (B) Representative flow cytometric analyses of the proportion of cTNT+ cells derived from different hPSC lines at day 24. (C) The hPSC-induced cells expressed α-actinin and cTNT indicated by immunofluorescent staining at day 24 of differentiation. Bar=50 μm. **Fig. S2**. Gene expression during chemically defined cardiac differentiation. Real-time PCR for markers of mesoderm (TBXT and MIXL1), cardiac mesoderm (MESP1), committed cardiac progenitors (NKX2-5, ISL1, and TBX5), and cardiomyocytes (MYH6, MYH7, and TNNT2). Values represent expression levels relative to the housekeeping gene GAPDH (n = 4). **Fig. S3**. Expression of SHOX2 and cTNT in SANLPCs. (A, B) Representative flow cytometric analyses of the proportion of SHOX2+/cTNT+ cells for control and PBC group at day 24. (n = 3). Mean ± SEM. *p < 0.05 by Student’s t test. (C, D) Immunofluorescent staining for SHOX2 and cTNT in PBC group, Bar=250 μm, (n = 3). Mean ± SEM. *p < 0.05 by Student’s t test. (E, F) Representative flow cytometric analyses of the proportion of SHOX2+/cTNT+ cells following metabolic selection or not at day 32 of differentiation. (n = 3). Mean ± SEM. *p < 0.05 by Student’s t test. **Fig. S4**. Generation of SANLPCs from different hPSC lines. (A) Relative expression of SAN gene for PBC group and control group in H9 and UiPS cell lines. (B-E) Representative flow cytometric analyses of the proportion of NKX2.5-/cTNT+ cells for control and PBC group at day 24 of differentiation in H9 and UiPS cell lines. Bar graph indicates average proportion of NKX2-5−/cTNT+ cells for control and PBC group from independent experiments (n = 3). Mean ± SEM. *p < 0.05 by Student’s t test. **Fig. S5**. The side-by-side comparisons between already available protocols (CHIR-protocol and BPM-protocol) and our current protocol. (A) Heatmap shows expression changes of genes related to SAN cells at day 24 via qPCR. (B-C) Representative flow cytometric analyses of the proportion of NKX2-5−/cTNT+ cells from three different protocols at day 32. **P ≤ 0.01 by Student’s t test (n = 3). All error bars represent the SD of three or four independent experiments. **Fig. S6**. Electrophysiological characterization of SANLCs generated by three different protocols. (A) Representative action potential recording using whole-cell patch from CHIR-protocol, BPM-protocol, and our current protocol. (B) Patch clamp recordings of induced cells from CHIR-protocol (15 cells), BPM-protocol (20 cells), and our current protocol (20 cells), demonstrating MDP, peak voltage, APA, APD90, APD50, and dV/dtmax (maximal rate of depolarization). (C) Bar graph indicates proportions of cardiomyocyte subtypes at day 32. MDP, maximum diastolic potential; APA, action potential amplitude; APD, action potential duration at different levels of repolarization (90% and 50%).

**Additional file 2: Table S1**. Primer sets for RT-PCR analysis. **Table S2**. Key resources table. **Table S3**. Electrophysiological characterization of cardiomyocytes generated from non-purified CDM3 group, purified CDM3 group, non-purified PBC group, and purified PBC group. Action potential (AP) recordings using whole cell patch clamp of hiPSC-derived cardiomyocytes from day 32 of differentiation. The AP characteristics used to classify cells into atrial-, nodal-, or ventricular-like. Includes MDP (maximum diastolic potential), peak voltage, APA (action potential amplitude), dV/dtmax (maximal rate of depolarization), and AP duration at different levels of repolarization (i.e., 90 or 50%). To determine the type of cardiomyocyte analyzed, subtypes were specified using the following characteristics: Ventricular-like, a negative maximum diastolic membrane potential (< -50 mV), a rapid AP upstroke, a long plateau phase, APA > 90 mV, and APD90/APD50 ratio < 1.4. Atrial-like, absence of a prominent plateau phase, a negative diastolic membrane potential (< -50 mV), and APD90/APD50 ratio > 1.7. Nodal-like, a more positive MDP, a slower AP upstroke, a prominent phase 4 depolarization, and APD90/APD50 ratio in between 1.4-1.7.

**Additional file 3: Video S1**. The CDM3 group showed sheet of beating cardiomyocytes.

**Additional file 4: Video S2**. The PBC group showed obvious multi-point pacing in the induced cells.

**Additional file 5: Video S3**. The PBC purification group showed multi-point pacing in the induced cells.

## Data Availability

All data generated or analyzed during this study are included in this published article (and its supplementary information files).

## Abbreviations

Aps: Action potentials
APD: Action potential duration
BMP: Bone morphogenic protein
CDM3: Chemically defined medium, 3 components
FGF: Fibroblast growth factor
GSK3: Glycogen synthase kinase 3
hPSCs: Human pluripotent stem cells
hESCs: Human embryonic pluripotent stem cells
HSF-iPSCs: Human skin fibroblasts-derived induced pluripotent stem cells
hPSC-CMs: Human pluripotent stem cell-derived cardiomyocytes
RT-PCR: Real-time polymerase chain reaction
RA: Retinoic acid
SAN: Sinoatrial node
SANLCs: Sinoatrial node-like cells
UiPSCs: Urine-derived induced pluripotent stem cells
VEGF: Vascular endothelial growth factor
WNT: WNT/β-catenin
