# Supplementary material for: Chemically defined and small molecules-based generation of sinoatrial node-like cells
Source: Stem Cell Res Ther. 2022 Apr 11;13:158. doi: 10.1186/s13287-022-02834-y (PMC8996538; doi:10.1186/s13287-022-02834-y)

Supplementary Figure 1

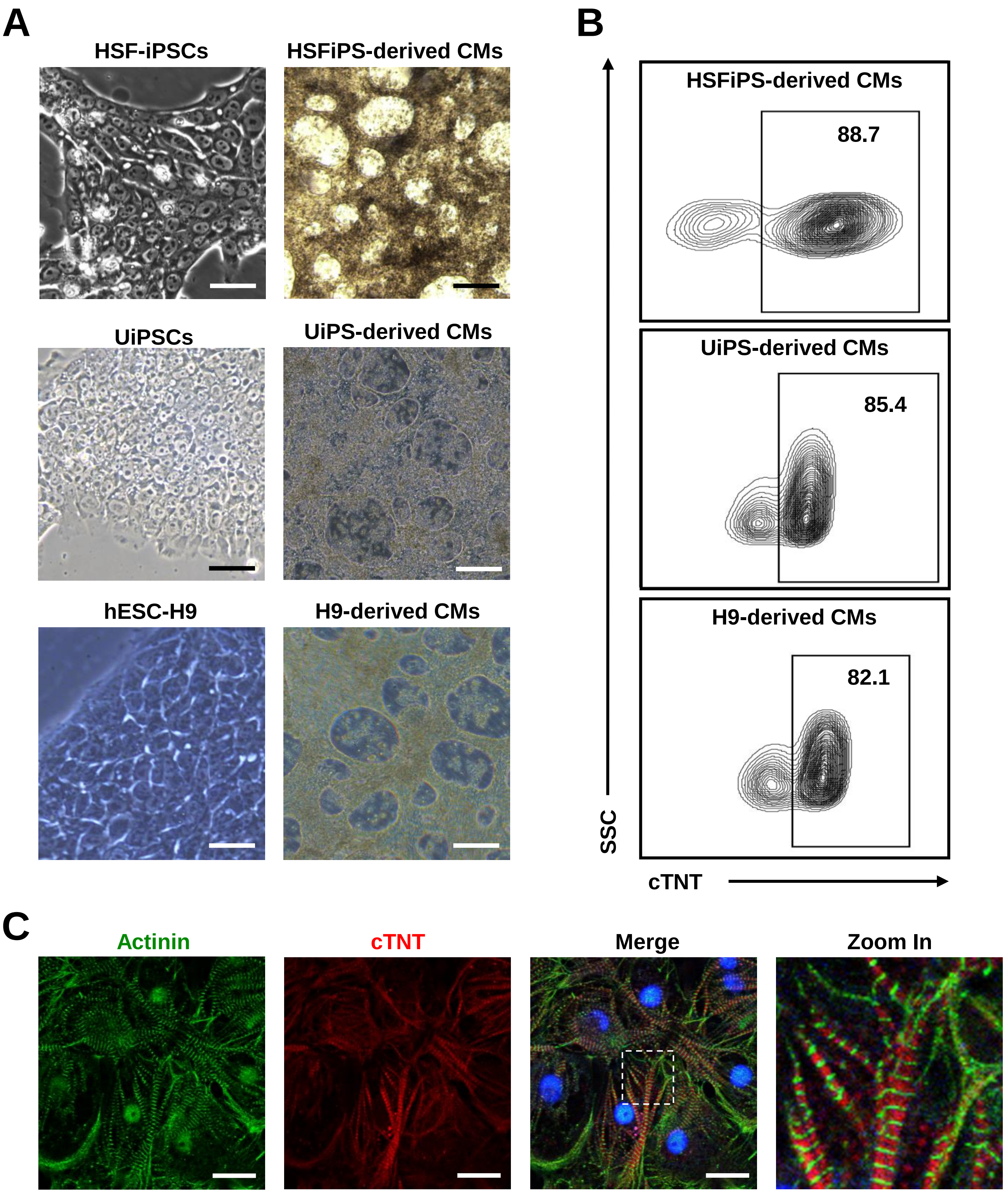

Supplementary Figure 2

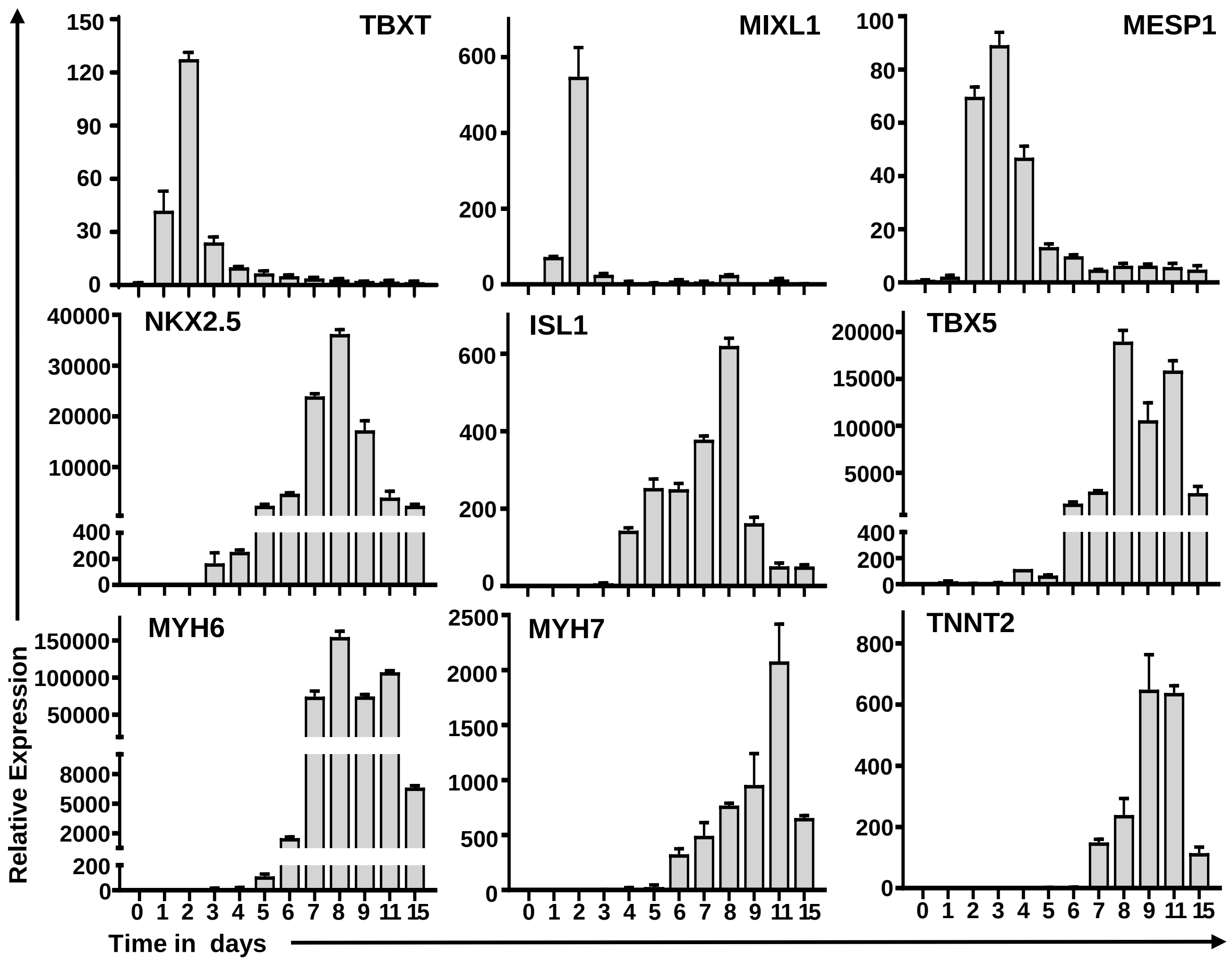

Supplementary Figure 3

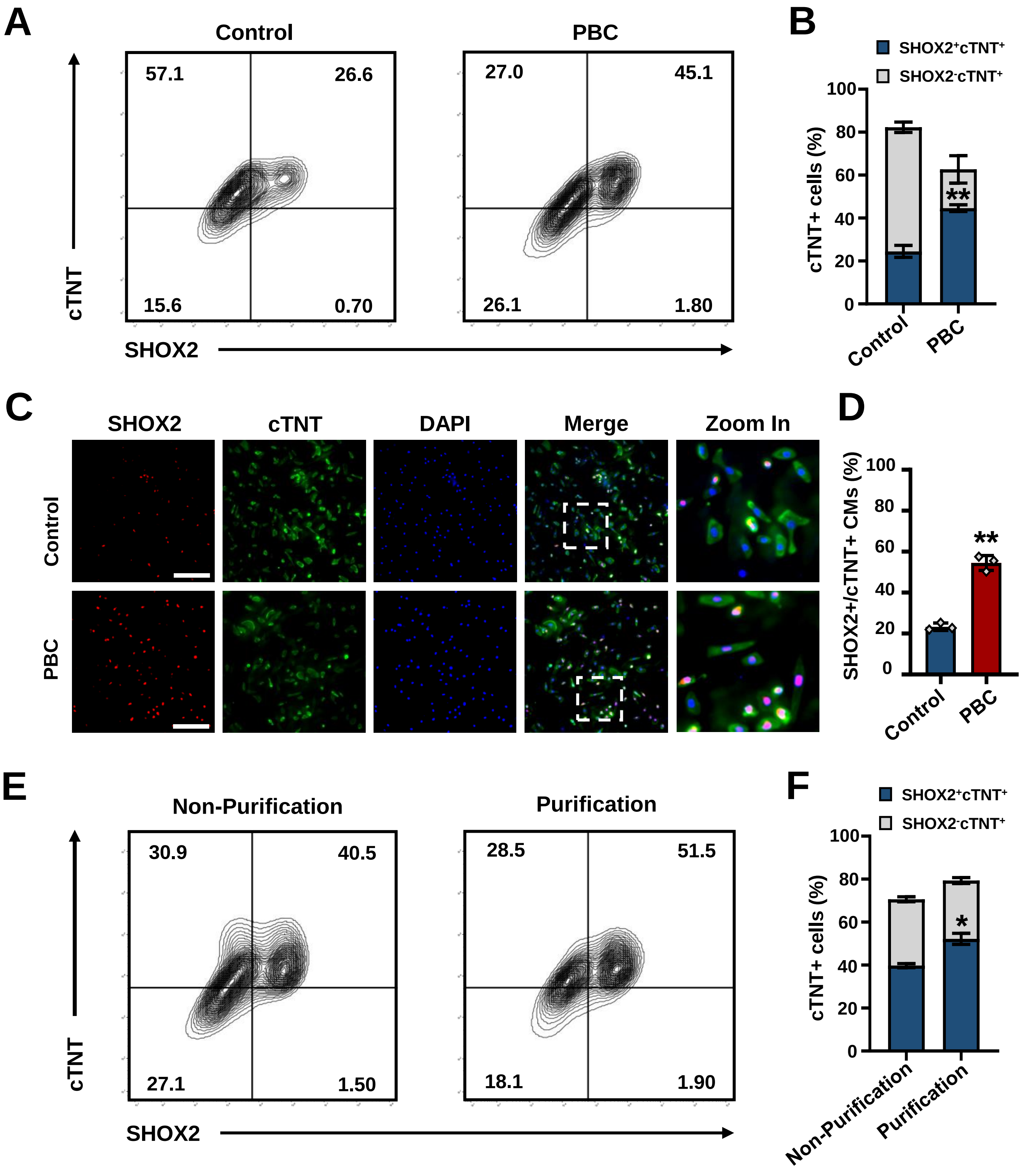

Supplementary Figure 4

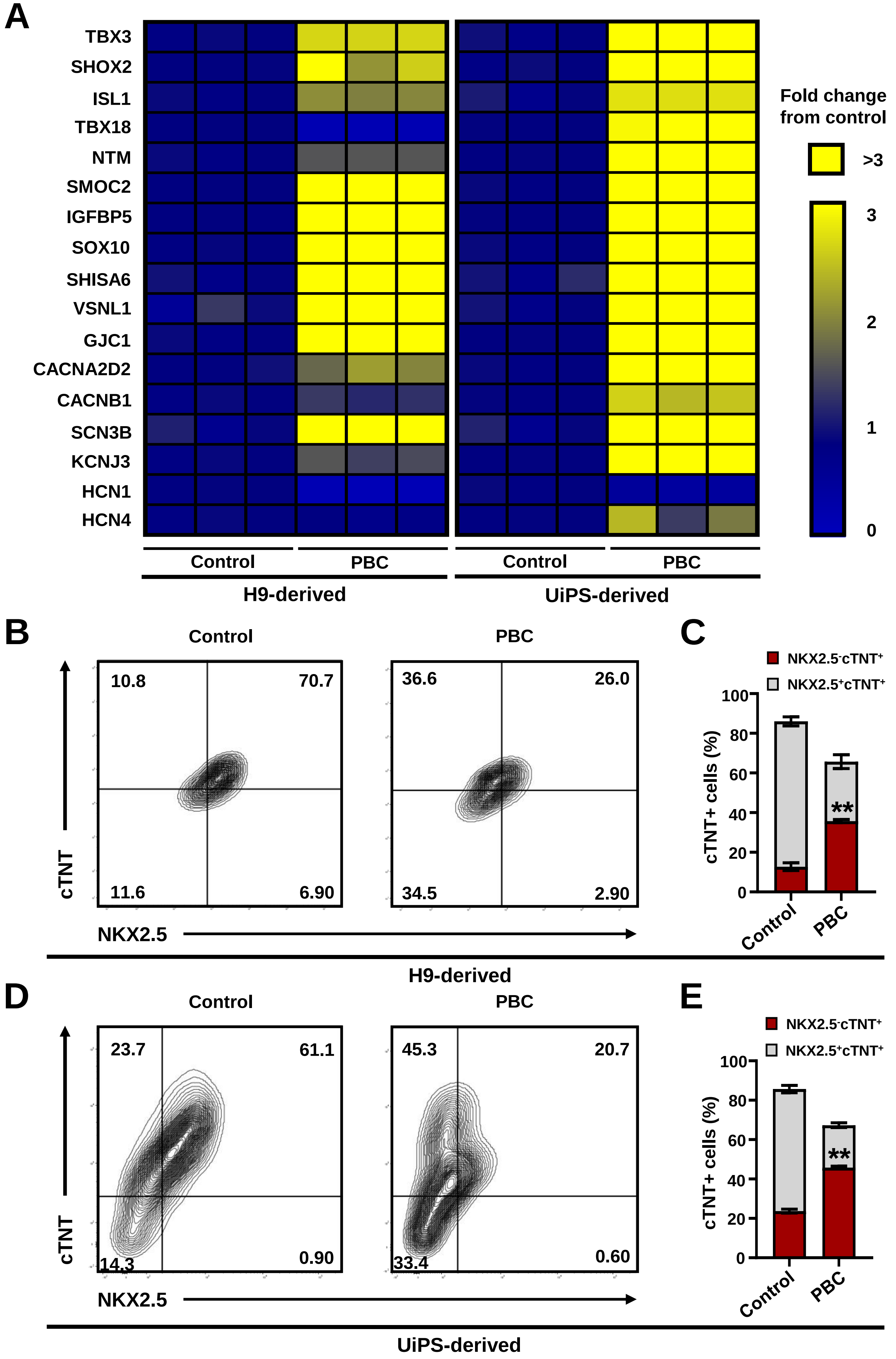

Supplementary Figure 5

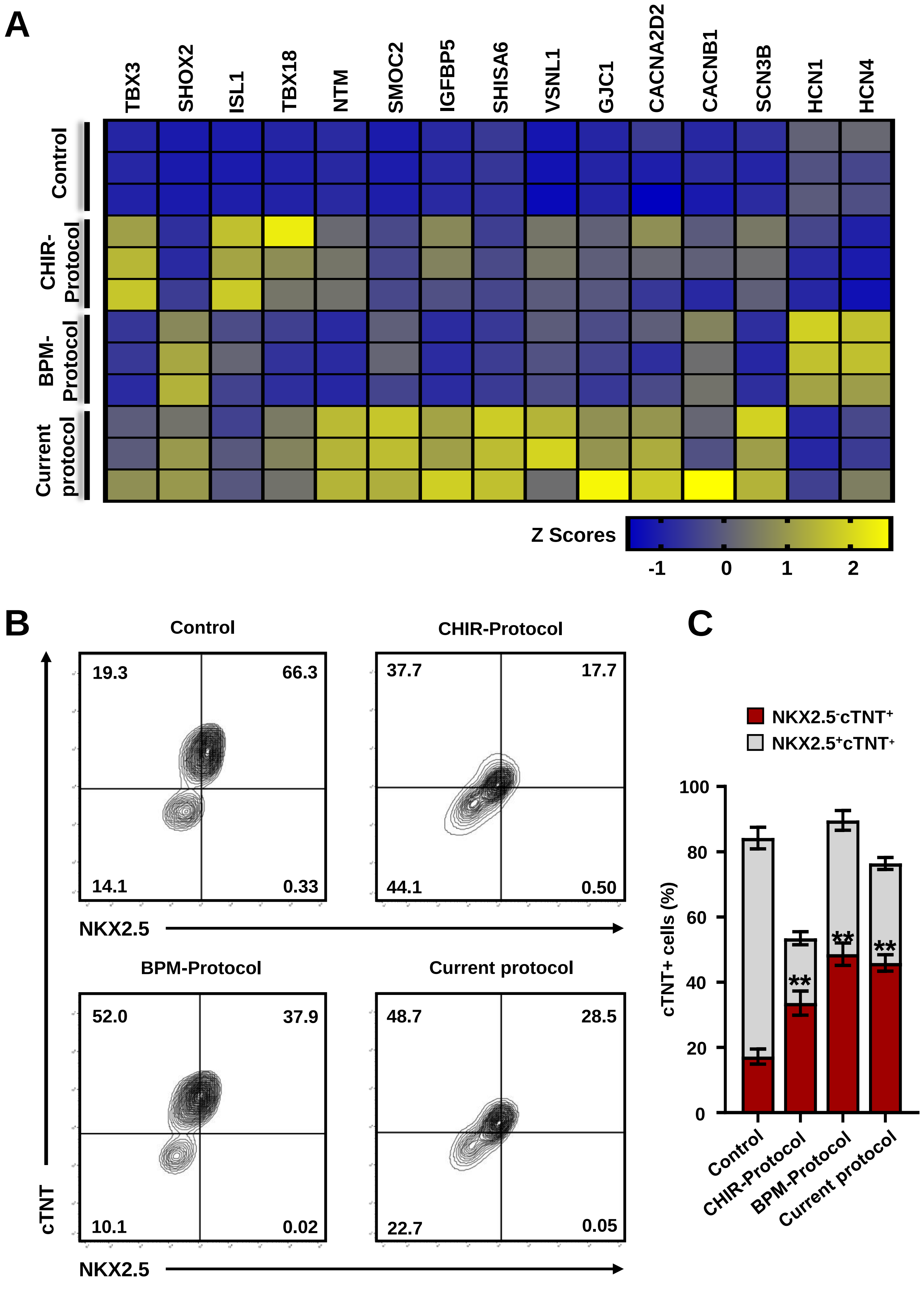

Supplementary Figure 6

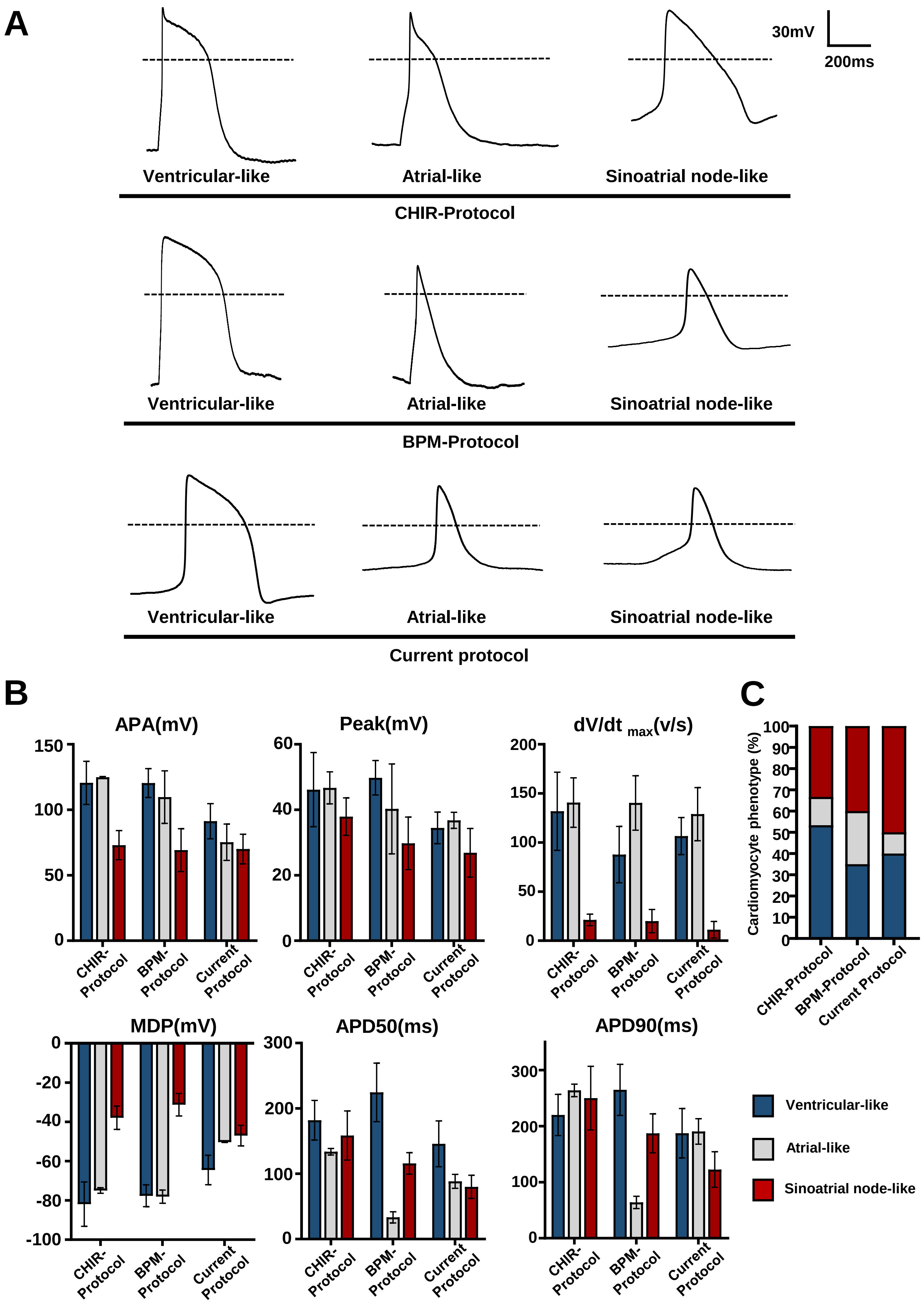

Supplement: Supplementary file 1 — Additional file 1: Fig. S1. Characterization of the hPSC-derived cardiomyocytes. (A) Morphological contrast image of different hPSC lines and hPSC-derived cardiomyocytes during chemically defined differentiation. (B) Representative flow cytometric analyses of the proportion of cTNT+ cells derived from different hPSC lines at day 24. (C) The hPSC-induced cells expressed α-actinin and cTNT indicated by immunofluorescent staining at day 24 of differentiation. Bar=50 μm. Fig. S2. Gene expression during chemically defined cardiac differentiation. Real-time PCR for markers of mesoderm (TBXT and MIXL1), cardiac mesoderm (MESP1), committed cardiac progenitors (NKX2-5, ISL1, and TBX5), and cardiomyocytes (MYH6, MYH7, and TNNT2). Values represent expression levels relative to the housekeeping gene GAPDH (n = 4). Fig. S3. Expression of SHOX2 and cTNT in SANLPCs. (A, B) Representative flow cytometric analyses of the proportion of SHOX2+/cTNT+ cells for control and PBC group at day 24. (n = 3). Mean ± SEM. *p < 0.05 by Student’s t test. (C, D) Immunofluorescent staining for SHOX2 and cTNT in PBC group, Bar=250 μm, (n = 3). Mean ± SEM. *p < 0.05 by Student’s t test. (E, F) Representative flow cytometric analyses of the proportion of SHOX2+/cTNT+ cells following metabolic selection or not at day 32 of differentiation. (n = 3). Mean ± SEM. *p < 0.05 by Student’s t test. Fig. S4. Generation of SANLPCs from different hPSC lines. (A) Relative expression of SAN gene for PBC group and control group in H9 and UiPS cell lines. (B-E) Representative flow cytometric analyses of the proportion of NKX2.5-/cTNT+ cells for control and PBC group at day 24 of differentiation in H9 and UiPS cell lines. Bar graph indicates average proportion of NKX2-5−/cTNT+ cells for control and PBC group from independent experiments (n = 3). Mean ± SEM. *p < 0.05 by Student’s t test. Fig. S5. The side-by-side comparisons between already available protocols (CHIR-protocol and BPM-protocol) and our current protocol. [file 13287_2022_2834_MOESM1_ESM.pdf]
