# Supplementary material for: Chemically defined and small molecules-based generation of sinoatrial node-like cells
Source: Stem Cell Res Ther. 2022 Apr 11;13:158. doi: 10.1186/s13287-022-02834-y (PMC8996538; doi:10.1186/s13287-022-02834-y)
Supplement: Supplementary file 2 — Additional file 2: Table S1. Primer sets for RT-PCR analysis. Table S2. Key resources table. Table S3. Electrophysiological characterization of cardiomyocytes generated from non-purified CDM3 group, purified CDM3 group, non-purified PBC group, and purified PBC group. Action potential (AP) recordings using whole cell patch clamp of hiPSC-derived cardiomyocytes from day 32 of differentiation. The AP characteristics used to classify cells into atrial-, nodal-, or ventricular-like. Includes MDP (maximum diastolic potential), peak voltage, APA (action potential amplitude), dV/dtmax (maximal rate of depolarization), and AP duration at different levels of repolarization (i.e., 90 or 50%). To determine the type of cardiomyocyte analyzed, subtypes were specified using the following characteristics: Ventricular-like, a negative maximum diastolic membrane potential (< -50 mV), a rapid AP upstroke, a long plateau phase, APA > 90 mV, and APD90/APD50 ratio < 1.4. Atrial-like, absence of a prominent plateau phase, a negative diastolic membrane potential (< -50 mV), and APD90/APD50 ratio > 1.7. Nodal-like, a more positive MDP, a slower AP upstroke, a prominent phase 4 depolarization, and APD90/APD50 ratio in between 1.4-1.7. [file 13287_2022_2834_MOESM2_ESM.docx]

**Suppl Table 1. Primer sets for RT-PCR analysis**

| **Gene name** | **Direction** | **Sequence（5’→3’）** |
| --- | --- | --- |
| CACNA1C | Forward | CAACACGGAGGTCAAGTTTAAG |
|  | Reverse | CATTGGCATTCATGTTGGCAT |
| CACNA2D2 | Forward | ATGGAAAACCGCAGACAAGAC |
|  | Reverse | CCGGGTAGTAGCGAGTGAC |
| CACNB1 | Forward | ACAATAATGACTGGTGGATCGG |
|  | Reverse | AACTGGAGTTATCGCCTGATTT |
| CPNE5 | Forward | GCACCGAAGTCATCGACAACA |
|  | Reverse | CAGGGGACCCCACAATCTC |
| GAPDH | Forward | GAGTCCACTGGCGTCTTCA |
|  | Reverse | TCTTGAGGCTGTTGTCATACTTC |
| GJA1 | Forward | CTTTCGTTGTAACACTCAGCAA |
|  | Reverse | CGCATCACATAGAACACATGAG |
| GJA5 | Forward | TCACTGTCCTCTTCATATTCCG |
|  | Reverse | GAATCGTATCACACCGGAAATC |
| GJC1 | Forward | GCTCACTGTTCTGATTGTCTTC |
|  | Reverse | CACAAATTTGCTTTGCTCATCG |
| HCN1 | Forward | CATGCCACCGCTTTAATCCAG |
|  | Reverse | ATTGTAGCCACCAGTTTCCGA |
| HCN4 | Forward | AGCTCATCTCCGCGTCTCA |
|  | Reverse | GCAAAGAACCTGAGGATGTCTT |
| IRX4 | Forward | CTATGGCAACTACGTGACCTAC |
|  | Reverse | CCGAACCATCCTTGGAATCAAA |
| ISL1 | Forward | ATGTGCGGAGTGTAATCAGTAT |
|  | Reverse | ATTTGATCCCGTACAACCTGAT |
| KCNE1 | Forward | ACTCGAACGACCCATTCAACG |
|  | Reverse | TTCAACGACATAGCACGACCT |
| KCNJ3 | Forward | GGCAGCGGTTCGTGGACAAG |
|  | Reverse | GTGGTGAAGAGGTCCGAGAGGTAG |
| KCNJ5 | Forward | GGAAGAGTTTGAAGTTGTGGTC |
|  | Reverse | GAGCACCTCTGTATCCATGTAG |
| KCNN4 | Forward | GCCATGCTGCTGCGTCTCTAC |
|  | Reverse | GGCGGAAGCGGACTTGATTGAG |
| KCNQ1 | Forward | CATTTCCATCATCGACCTCATC |
|  | Reverse | CGATGTACAGGGTGGTTATCAG |
| MESP1 | Forward | AGCCCAAGTGACAAGGGACAACT |
|  | Reverse | AAGGAACCACTTCGAAGGTGCTGA |
| MIXL1 | Forward | ACTGAAACGAAATGTCTGAAGC |
|  | Reverse | CAGCTTTGAACCAATGTCTTCA |
| MSX2 | Forward | GGTCAAAATCTGGTTCCAGAAC |
|  | Reverse | TAGGTTTTGCAGCCATTTTCAG |
| MYH11 | Forward | GACCTCTTTCGTTCCTTCTAGA |
|  | Reverse | CTTGGTTCCATTGAAGTCTGC |
| MYH6 | Forward | GCCCTTTGACATTCGCACTG |
|  | Reverse | GGTTTCAGCAATGACCTTGCC |
| MYH7 | Forward | TCACCAACAACCCCTACGATT |
|  | Reverse | CTCCTCAGCGTCATCAATGGA |
| MYL2 | Forward | TCCGGGTCCAATTAACTTTACT |
|  | Reverse | CAAACACTTTGAATGCGTTGAG |
| MYL7 | Forward | GAACAAGGATGAGTTCAAGCAG |
|  | Reverse | TGATGATGTAGCACAGTGACTT |
| NKX2.5 | Forward | CAAGTGTGCGTCTGCCTTT |
|  | Reverse | CAGCTCTTTCTTTTCGGCTCTA |
| NPPA | Forward | CAACGCAGACCTGATGGATTT |
|  | Reverse | AGCCCCCGCTTCTTCATTC |
| NR2F2 | Forward | GTGTGCTTTGGAAGAATACGTT |
|  | Reverse | CCAAACGGACGAAAAACAATTG |
| PDGFRa | Forward | GAAAATGAAAAGGTTGTGCAGC |
|  | Reverse | CTCTTCTTCAGACATGGGGTAC |
| PDGFRb | Forward | AGCACCTTCGTTCTGACCTG |
|  | Reverse | TATTCTCCCGTGTCTAGCCCA |
| PECAM1 | Forward | TCGTGGTCAACATAACAGAACT |
|  | Reverse | TTGAGTCTGTGACACAATCGTA |
| REC114 | Forward | CGCCTGTTTCGAGTACAGTTC |
|  | Reverse | TGCCGTGGGACACTCTTTG |
| SCN3B | Forward | GCTTCTCTCGTGCTTATCTACT |
|  | Reverse | ATAAGGAAATCTTTACCGCCCT |
| SCN5A | Forward | ATCTTCACAGGCGAGTGTATTG |
|  | Reverse | ACAACCACGAAGTCGAAGATAT |
| SHOX2 | Forward | ATAAAGGTGTTCTCATAGGGGC |
|  | Reverse | ATCCTTAAAGCACCTACGTTGA |
| SMOC2 | Forward | ATGACGACGGCACCTACAG |
|  | Reverse | TCGCGTTGGGGTAACTTTTCA |
| SOX10 | Forward | CCTCACAGATCGCCTACACC |
|  | Reverse | CATATAGGAGAAGGCCGAGTAGA |
| TBX18 | Forward | AAAGACTGTGGAGACGATCTTT |
|  | Reverse | AATCTGCTGATTCTGATAGGCA |
| TBX2 | Forward | GCTGACGATTGCCGCTATAAG |
|  | Reverse | GGCTGTCTGGGTGGATGTA |
| TBX3 | Forward | CATGAGAGATCCGGTCATTCC |
|  | Reverse | CAATTGATCCATGATCGGCTTG |
| TBX5 | Forward | CAGTTACAAAGTGAAGGTGACG |
|  | Reverse | TGCGAATTTGTATCTGTGATCG |
| TBXT | Forward | TAAGTATGAGCCTCGAATCCAC |
|  | Reverse | GATCTCCTCGTTCTGATAAGCA |
| TCF21 | Forward | GGCTAACGACAAATACGAGAAC |
|  | Reverse | ACTTCTTTCAGGTCACTCTCG |
| TNNT2 | Forward | TTCTCTCAAAGACAGGATCGAG |
|  | Reverse | GTTGGACAAAGCCTTCTTCTTC |
| UPK3b | Forward | TGCCCTACACACCACAGATAA |
|  | Reverse | GCAAGCCCATCGAAGACAC |
| VIM | Forward | GACGCCATCAACACCGAGTT |
|  | Reverse | CTTTGTCGTTGGTTAGCTGGT |
| VSNL1 | Forward | TGCCTTCCGAACCTTCGAC |
|  | Reverse | CATCTCCACTCGGGTGATCT |
| WT1 | Forward | ATTGACAAAGTCAGCCCCTATG |
|  | Reverse | GAGAAGAGTCAAGATAGGCAGG |

**Suppl Table 2. KEY RESOURCES TABLE**

| **REAGENT or RESOURCE** | **SOURCE** | **IDENTIFIER** |
| --- | --- | --- |
| **Antibodies** | | |
| Rabbit anti-human α-Actinin antibody | Abcam | Cat# ab137346 |
| Mouse anti-human cTNT antibody | Thermo Fisher Scientific | Cat# MA5-12960  RRID: AB_11000742 |
| Rabbit anti-human cTNT antibody | Proteintech | Cat# 15513-1-AP,  RRID: AB_2206563) |
| Rabbit anti-human NKX2.5 antibody | Abcam | Cat# ab97355  RRID: AB_10680260 |
| Mouse anti-human SHOX2 antibody | Abcam | Cat# ab55740  RRID: AB_945451 |
| **Signaling pathway agonists and inhibitors** | | |
| CHIR99021 | Sigma-Aldrich | Cat# SML1046 |
| Wnt-C59 | Selleck Chemicals | Cat# S7037 |
| PD173074 | Selleck Chemicals | Cat# S1264 |
| BMS-189453 | Sigma-Aldrich | Cat# SML1046 |
| Thiazovivin | Selleck Chemicals | Cat# S1459 |
| **Cell culture related reagents** | | |
| PSCeasy® medium | Cellapy | Cat# CA1014500 |
| CardioEasy® medium | Cellapy | Cat# CA2004500 |
| RPMI 1640 medium without d-glucose | Life Technologies | Cat# 11879020 |
| CardioEasy® cardiomyocyte dissociation buffer Ⅰ | Cellapy | Cat# CA2011100 |
| CardioEasy® cardiomyocyte dissociation buffer Ⅱ | Cellapy | Cat# CA2012100 |
| **Chemicals, Peptides, and Recombinant Proteins** | | |
| Bovine serum albumin | Sigma-Aldrich | Cat# A2153 |
| Matrigel | Corning | Cat# 354277 |
| L-ascorbic acid 2-phosphate | Sigma-Aldrich | Cat# 66170-10-3 |
| O. sativa–derived recombinant human albumin | Sigma-Aldrich | Cat# A0237 |
| Sodium dl-lactate | Sigma-Aldrich | Cat# L4263 |
| Triton X-100 | Invitrogen | Cat# 85111 |
| 4% Paraformaldehyde Fix Solution | Beyotime | Cat# P0099 |
| DAPI | Cell Signaling Technology | Cat# 4083 |
| 0.5 mM EDTA | Cellapy | Cat# CA3001500 |
| **Critical Commercial Assays** | | |
| TRIzol Reagent | Invitrogen | Cat# 15596026 |
| Prime-ScriptTM reverse transcription kit | TaKaRa | Cat# RR037A |
| TB Green™ Premix Ex Taq™ II | TaKaRa | Cat# RR820A |
| **Experimental Models: Cell Lines** | | |
| HSF-iPSCs | OSINGLAY BIO | HNF-P30-P11 |
| hESCs-H9 | WiCell Research Institute, Inc. | WA09 |
| UiPSCs | Cellapy | CA1002008 |

**Suppl Table 3**

| **Non-purified CDM3 group** | | | | | | | |
| --- | --- | --- | --- | --- | --- | --- | --- |
| APs  (n=20) | APA  (mA) | Peak  (mV) | dv/dt_max_  (V/s) | MDP  (mV) | APD90  (ms) | APD50  (ms) | APD90/APD50 |
| Ventricular-like  (n = 15) | 102.6  ±14.9 | 35.3  ±8.3 | 132.4  ±37.6 | -61.3  ±9.5 | 160.5  ±44.8 | 129.5  ±38.5 | 1.25  ±0.1 |
| Atrial-like  (n = 3) | 105.3  ±27.3 | 37.9  ±3.7 | 143.8  ±32.4 | -63.6  ±15.3 | 198.5  ±54.9 | 91.3  ±17.9 | 2.15  ±0.2 |
| Nodal-like  (n = 2) | 78.4  ±4.7 | 29.1  ±8.9 | 7.9  ±0.9 | -48.9  ±4.8 | 177.7  ±12.6 | 112.9  ±3.8 | 1.57  ±0.1 |
| **Purified CDM3 group** | | | | | | | |
| APs  (n=20) | APA  (mA) | Peak  (mV) | dv/dt_max_  (V/s) | MDP  (mV) | APD90  (ms) | APD50  (ms) | APD90/APD50 |
| Ventricular-like  (n = 14) | 124.7  ±12.9 | 47.9  ±6.2 | 102.4  ±24.3 | -77.0  ±8.7 | 214.2  ±58.1 | 170.8  ±51.2 | 1.25  ±0.1 |
| Atrial-like  (n = 3) | 130.2  ±9.9 | 47.1  ±7.1 | 119.6  ±33.7 | -82.5  ±4.9 | 169.7  ±15.3 | 79.4  ±4.7 | 2.14  ±0.2 |
| Nodal-like  (n = 3) | 70.0  ±19.6 | 30.4  ±5.7 | 8.7  ±3.4 | -36.8  ±19.3 | 181.4  ±71.6 | 112.0  ±37.3 | 1.59  ±0.2 |
| **Non-purified PBC group** | | | | | | | |
| APs  (n=12) | APA  (mA) | Peak  (mV) | dv/dt_max_  (V/s) | MDP  (mV) | APD90  (ms) | APD50  (ms) | APD90/APD50 |
| Ventricular-like  (n = 5) | 107.6  ±14.5 | 34.9  ±9.9 | 101.4  ±21.4 | -69.9  ±10.5 | 300.6  ±39.7 | 247.2  ±29.5 | 1.20  ±0.1 |
| Atrial-like  (n = 2) | 55.4  ±8.2 | 21.2  ±3.7 | 106.5  ±0.5 | -36.5  ±6.9 | 444.3  ±102.2 | 191.3  ±32.0 | 2.04  ±0.1 |
| Nodal-like  (n = 5) | 68.7  ±5.1 | 30.5  ±8.9 | 22.0  ±8.9 | -42.5  ±4.6 | 259.3  ±39.5 | 166.7  ±28.2 | 1.56  ±0.1 |
| **Purified PBC group** | | | | | | | |
| APs  (n=20) | APA  (mA) | Peak  (mV) | dv/dt_max_  (V/s) | MDP  (mV) | APD90  (ms) | APD50  (ms) | APD90/APD50 |
| Ventricular-like  (n = 8) | 91.3  ±13.4 | 34.5  ±4.8 | 106.6  ±18.9 | -64.5  ±7.5 | 187.2  ±44.1 | 145.8  ±35.1 | 1.29  ±0.1 |
| Atrial-like  (n = 2) | 75.2  ±13.9 | 36.8  ±2.5 | 128.9  ±27.1 | -50.3  ±0.2 | 190.5  ±22.7 | 88.3  ±10.7 | 2.16  ±0.1 |
| Nodal-like  (n = 10) | 70.0  ±11.3 | 26.9  ±7.4 | 11.2  ±8.4 | -47.0  ±5.2 | 122.5  ±31.7 | 79.9  ±17.7 | 1.52  ±0.1 |

**Table S3.** Electrophysiological characterization of cardiomyocytes generated from non-purified CDM3 group, purified CDM3 group, non-purified PBC group, and purified PBC group. Action potential (AP) recordings using whole cell patch clamp of hiPSC-derived cardiomyocytes from day 32 of differentiation. The AP characteristics used to classify cells into atrial-, nodal-, or ventricular-like. Includes MDP (maximum diastolic potential), peak voltage, APA (action potential amplitude), dV/dtmax (maximal rate of depolarization), and AP duration at different levels of repolarization (i.e., 90 or 50%). To determine the type of cardiomyocyte analyzed, subtypes were specified using the following characteristics: Ventricular-like, a negative maximum diastolic membrane potential (< -50 mV), a rapid AP upstroke, a long plateau phase, APA > 90 mV, and APD90/APD50 ratio < 1.4. Atrial-like, absence of a prominent plateau phase, a negative diastolic membrane potential (< -50 mV), and APD90/APD50 ratio > 1.7. Nodal-like, a more positive MDP, a slower AP upstroke, a prominent phase 4 depolarization, and APD90/APD50 ratio in between 1.4-1.7.
